# Transferability Based on Drug Structure Similarity in the Automatic Classification of Noncompliant Drug Use on Social Media: Natural Language Processing Approach

**DOI:** 10.2196/44870

**Published:** 2023-05-03

**Authors:** Tomohiro Nishiyama, Shuntaro Yada, Shoko Wakamiya, Satoko Hori, Eiji Aramaki

**Affiliations:** 1 Department of Information Science Nara Institute of Science and Technology Ikoma Japan; 2 Division of Drug Informatics Keio University Faculty of Pharmacy Tokyo Japan

**Keywords:** data mining, machine learning, medication noncompliance, natural language processing, pharmacovigilance, transfer learning, text classification

## Abstract

**Background:**

Medication noncompliance is a critical issue because of the increased number of drugs sold on the web. Web-based drug distribution is difficult to control, causing problems such as drug noncompliance and abuse. The existing medication compliance surveys lack completeness because it is impossible to cover patients who do not go to the hospital or provide accurate information to their doctors, so a social media–based approach is being explored to collect information about drug use. Social media data, which includes information on drug usage by users, can be used to detect drug abuse and medication compliance in patients.

**Objective:**

This study aimed to assess how the structural similarity of drugs affects the efficiency of machine learning models for text classification of drug noncompliance.

**Methods:**

This study analyzed 22,022 tweets about 20 different drugs. The tweets were labeled as either noncompliant use or mention, noncompliant sales, general use, or general mention. The study compares 2 methods for training machine learning models for text classification: single-sub-corpus transfer learning, in which a model is trained on tweets about a single drug and then tested on tweets about other drugs, and multi-sub-corpus incremental learning, in which models are trained on tweets about drugs in order of their structural similarity. The performance of a machine learning model trained on a single subcorpus (a data set of tweets about a specific category of drugs) was compared to the performance of a model trained on multiple subcorpora (data sets of tweets about multiple categories of drugs).

**Results:**

The results showed that the performance of the model trained on a single subcorpus varied depending on the specific drug used for training. The Tanimoto similarity (a measure of the structural similarity between compounds) was weakly correlated with the classification results. The model trained by transfer learning a corpus of drugs with close structural similarity performed better than the model trained by randomly adding a subcorpus when the number of subcorpora was small.

**Conclusions:**

The results suggest that structural similarity improves the classification performance of messages about unknown drugs if the drugs in the training corpus are few. On the other hand, this indicates that there is little need to consider the influence of the Tanimoto structural similarity if a sufficient variety of drugs are ensured.

## Introduction

Medication compliance, a type of health literacy defined as a patient’s use of medications [1], is a critical issue because an increased number of drugs have been sold on the web. Web-based drug distribution is difficult to control, causing problems such as drug noncompliance and abuse [2]. Thus, the importance of medication compliance surveys, such as what kinds of medications tend to be abused, is increasing. However, medication compliance surveys are unreliable because it is impossible to cover patients who do not go to the hospital or provide accurate information to their doctors. This situation motivates a social media–based approach because some patients provide information about drug usage. Therefore, social media is attracting attention for collecting knowledge about drug use information [3-6].

We attempted to use social media to catch medication compliance, people’s understanding of drugs, and other health-related information to understand the patients’ medication status and knowledge. This information should be useful as an early signal for the dissemination and understanding of regulations and safety information from drug regulatory authorities and drug suppliers. There are many potential ways to use social media; drug regulatory authorities and drug suppliers might detect specific drugs that are likely to be misused by automatically classifying comments. Some studies have linked other compliant use statistics to the number of medication noncompliance tweets, and real-time message collection might be expected to expedite drug regulation [7]. Ru et al [8] mentioned some patients reported serendipitous new indications for the drugs they were using for comorbidity, which is valuable information for drug repositioning on social media sites.

In addition, social media is expected to be one of the methods to catch the voice of patients for the supplement of traditional questionnaire-based surveys. There are 2 methods of information extraction from social media, which is manual annotation and machine learning method. As research examples of manual annotation, Sinnenberg et al [9] and Golder et al [6] used it in tweets to categorize the statements about drugs for certain kinds of drugs such as drugs for cardiovascular disease or statins. Gkotsis et al [10] used it in Reddit posts to understand the characteristics of users diagnosed with dementia. Wexler et al [11] and Beusterien et al [12] used manual coding to study certain forums related to health. As examples of machine learning methods, Mao et al [13] studied how users discussed the side effects of aromatase inhibitors and concerns about risk-benefit balance. Burkhardt et al [14] used a semisupervised learning method to detect side effects reported in tweets. Rastegar-Mojarad et al [15] and Zhao and Yang [16] use machine learning approaches to detect potential candidates for drug repositioning. Weissenbacher et al [17] created an ensemble learning classifier that can identify tweets mentioning drugs and dietary supplements. Sarker and Gonzalez [18] created a corpus to identify drugs on Twitter, with potential applications for monitoring drug efficacy, side effects, and user sentiment toward drugs.

Moreover, some attempts have been made to detect drug abuse and medication compliance in patients [19-26]. Abdellaoui et al [24] performed tweet classification using a topic model for 2 drugs: *escitalopram* and *aripiprazole*. Weinssenbacher et al [19] proposed a method for detecting drug dosage changes in noncompliant patients. Bigeard et al [26] attempted to detect drug misuse and found that using Anatomical Therapeutic Chemical (ATC) codes and text in the classification task improved the accuracy of misuse detection. However, the existing methods do not fully use information on drugs, such as the structure of the active ingredients.

**Figure 1:**
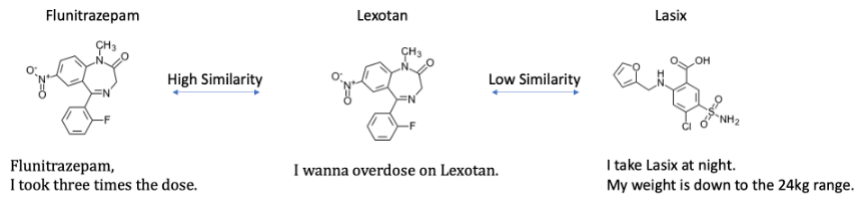
Our approach, transfer learning based on chemical structures, assumes similarly structured corpora are transferable.

In our approach, the method of developing a corpus is practically a big issue because the corpus highly depends on the drug type. This means that we are suffering from covering all drug types because the nature of the text varies widely from drug to drug. As shown in Figure 1, medication noncompliance tweets of drugs classified as sleeping pills and anxiolytics, such as *Lexotan*, stand out as overdosed (Figure 1, left and middle). On the other hand, diuretics such as *Lasix* stand out in tweets suggesting that they are used for dieting (Figure 1, right). Thus, the messages differed for each drug type. This makes classification more difficult and results in lower accuracy. In such cases, supervised learning is optimal for classifying tweets about various drugs with high accuracy [19,27-29], and a corpus for each drug is necessary. However, building such a corpus is time- and money-consuming.

To make use of the limited data, we attempted transfer learning to reuse the data for training, in which a corpus created for a specific drug is used for other medications. We used drug structural similarity as a training method. Drugs with similar chemical structures are likely to have similar mechanisms of action and can be used for similar purposes. Specifically, Martin et al [30] demonstrated that structurally similar drugs have similar mechanisms of action. Meyer et al [31] used the structural information of a drug to predict its usage. Therefore, it is conceivable that tweets which mention similar drugs about medication noncompliance are also expected to be similar [19,27-29]. For example, the drug *Flunitrazepam*, which has a chemical structure similar to *Lexotan*, is likely to be used effectively as training data (Figure 1).

Therefore, we performed transfer learning of a corpus for drugs with similar chemical structures. To conduct transfer learning, we prepared a MediA corpus data set to monitor medication noncompliance. In this corpus, we defined noncompliance as a message that indicates the speaker’s incorrect perception of handling a drug. Specifically, messages showing noncompliance were labeled as “*Noncompliant use or mention* (*NC-u/m*),” among which messages about buying and selling were marked as “*Noncompliant sales* (*NC-s*),” and messages about medication that was not noncompliant were labeled as “*General use* (*G-u*).” All other messages were labeled as “*General mention* (*G-m*).”

The contributions of this study are as follows:

1. Construction of a corpus labeled for medication noncompliance.
2. We propose a transfer learning method that uses chemical structures. Language processing can use these features, but this has not yet been addressed in the existing research.

In this study, we performed transfer learning to classify tweets about different drugs using a model trained on tweets about specific drugs in our corpus and discussed the results in terms of drug characteristics. In addition, we focused on the chemical structure of the drugs and verified their learning efficiency using the similarity of chemical structures. These results suggest that learning efficiency improves with limited drug data.

## Methods

### Materials

The corpus consisted of 22,022 tweets referring to 20 drugs labeled according to noncompliance. The 20 drugs included *Loxonin* (*Loxoprofen*) and *Voltaren* (*Diclofenac*) for pain relief; *Myslee* (*Zolpidem*), *Flunitrazepam*, *Lexotan* (*Bromazepam*), *Lunesta* (*Eszopiclone*), *Depas* (*Etizolam*), and *Belsomra* (*Suvorexant*) for sleep and antianxiety; *Paxil* (*Paroxetine*), *Lexapro* (*Escitalopram*), *Sertraline*, *Abilify* (*Aripiprazole*), *Contomin* (*Chlorpromazine*), *Zyprexa* (*Olanzapine*), and *Risperdal* (*Risperidone*) for antipsychotic drugs; *Restamine* (*Diphenhydramine*) for antiallergic drugs; *Medicon* (*Dextromethorphan*) for a cough suppressant; *Zithromax* (*Azithromycin*) for an antibiotic; *Metformin* for diabetes treatment; and *Lasix* (*Furosemide*) for a diuretic. *Flunitrazepam, Sertraline,* and *Metformin* are generic names. The words used as drug queries were “*Loxonin*,” “*Voltaren*,” “*Myslee*,” “*Flunitrazepam*,” “*Lexotan*,” “*Lunesta*,” “*Depas*,” “*Belsomra*,” “*Paxil*,” “*Lexapro*,” “*Sertraline*,” “*Abilify*,” “*Contomin*,” “*Zyprexa*,” “*Risperdal*,” “*Restamin*,” “*Medicon*,” “*Zithromax*,” “*Metformin*,” and “*Lasix,*” respectively. The 20 drugs were selected based on the following criteria: (1) they are commonly prescribed drugs or used as over-the-counter drugs, and the query is a brand name or generic name, and (2) the number of tweets in the past 3 years must be more than 1000 to ensure sufficient volume. We manually selected the 20 drug queries with less advertisements and promotional messages. Tweets were collected using 20 drug queries from January 1, 2017, to December 31, 2020, before random sampling 1000 tweets for each drug.

In this corpus, noncompliance was defined as a tweet that could be read as the writer’s incorrect perception of handling a drug and was categorized into four types: *noncompliant use or mention, noncompliant sales, general use*, and *general mention,* as shown in Textbox 1. Specifically, tweets that could be read as noncompliant were marked as “*Noncompliant use or mention* (*NC-u/m*),” tweets related to buying and selling were labeled as “*Noncompliant sales* (*NC-s*),” tweets related to medication that were not noncompliant were labeled as “*General use* (*G-u*),” and tweets other than those are marked as “*General mention* (*G-m*).” Even if it is not a definitive noncompliance, a statement, including exaggeration, is defined as noncompliance. For instance, we judged the first example is doubted as noncompliance because it is doubted that the user took more drugs than he needed. The reason why we set the criteria if the statement is possibly doubted as noncompliance is for capturing the small signal of noncompliance. Textbox 1 presents a part of examples of the MediA corpus. The detailed examples and guidelines of the corpus are shown in Multimedia Appendix 1.

As for the annotation results, of 22,022 cases, 4630 were “*NC-u/m*,” 1577 were “*NC-s*,” 8326 were “*G-u*,” and 7489 were “*G-m*.” The Cohen kappa coefficient was 0.695, indicating a substantial agreement [32]. Annotation was performed by 3 persons, 1 with pharmacological knowledge and 2 with sufficient experience in annotating biomedical documents.

**Examples of MediA corpus.**

**Noncompliant use or mention (NC-u/m)**

- デパス多めに飲んだ (I took more *Depas*)
- デパスの処方やめるって言われたら生きていかれないと思う (I don’t think I could live with myself if they told me to stop prescribing *Depas*)
- 眠剤とデパスに依存症になって，カー！！デパス効いてきてふわふわ気持ちいい (I’ve become addicted to sleeping pills and *Depas*, I feel lightheaded and comfortable as *Depas* is working)

**Noncompliant sales** **(NC-s)**

- レクサプロ⋅ジェネリック抗うつ剤のレクサプロジェネリック医療品でうつ病や、パニック障害、対人恐怖症、不安障害に有効的です 20 mg × 200錠 ¥14,000 (US $104) (*Lexapro* Generic: Antidepressant *Lexapro* generic medical product effective for depression, panic disorder, interpersonal phobia, anxiety disorder 20 mg × 200 tablets ¥14,000 [US $104])

**General use (G-u)**

- リスパダール飲んだ
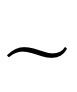
ぞ。(I took *Risperdal*)

**General mention (G-m)**

- ラシックスなしで着順上げながら三冠完走って只者じゃなかったね (He was not a simpleton to finish the Triple Crown without *Lasix* while improving his finishing order)

### Experiment Design

We conducted an experiment to compare the learning efficiency of text classification for drug noncompliance. The objective of the study was to clarify how the structural similarity of drugs affects the learning models for the text classification of drugs. The motivation for this experiment was as follows: Each active ingredient in a drug has a unique structure. We hypothesized that texts whose drugs had similar chemical structures would be similar. Therefore, we expected that the similarity of the chemical structures of the drugs would help train a model for text classification.

There are 2 methods, single-subcorpus transfer learning and multi-subcorpus incremental learning, which we designed in this study. In the single subcorpus transfer learning, we classified tweets mentioning other drug queries using a model trained on every single drug. We compared the structural similarity and model classification performance to investigate the relationship between the similarity and classification metrics. In multi-subcorpus incremental learning, we checked the classification performance of models trained by tweets mentioning the drug query selected in order of similarity. We demonstrate the usefulness of similarity by comparing it with a randomly trained model.

This learning method comes from the idea of the following usage: When pharmaceutical companies and authorities use social media to catch the potential signal from social media of medication noncompliance for each drug, they use models trained. To evaluate medication noncompliance in a low-resource language, it is necessary to begin with the creation of a corpus. However, the size of the corpus and the drugs selected should be limited because corpus creation is costly. A certain drug corpus is essential if it can be used for other drug texts by transfer learning.

### Classifier

Experiments were conducted using bidirectional encoder representations from transformer (BERT)–based classifiers. A pretrained model of BERT (we adopted the pretrained BERT model “bert-base-Japanese-whole-word-masking” downloaded from Huggingface Hub [33]) using Japanese Wikipedia was exploited and fine-tuned using the MediA corpus. The model consisted of 12 layers, 768-dimensional hidden layers, and 12 attention heads. We used the CLS token of the last layer to classify texts. A classification task was performed to evaluate the usefulness of this corpus.

We used BERT as a text classification model since the BERT model achieved better results compared to light-weighted models such as Word2vec embedding+LSTM and N-gram+traditional models. Specifically, Al-Garadi et al [34] compared BERT and the model used Twitter Glove embeddings + BiLSTM model in tweet classification of drug use and showed BERT was a better performance than the BiLSTM-based model. Tassone et al [35] also compared the model of BERT and XGBoost for tweet classification and showed BERT obtained better results.

#### Initial Settings

The labeled data set of the MediA corpus was divided into 3 parts in an 80:10:10 ratio; the larger set was used for training and the 2 smaller sets for development and testing. For all the models trained in this study, the training was stopped at the point where the validation loss was the smallest.

#### Single Subcorpus Transfer Learning

Let us say that a pair of drug queries *i* and *j* are given labels of tweets *D_j_* that mention drug query *j* predicted by using model *M_i_* built with tweets *D_i_* that mention drug query *i*. In the case of *i* ≠ *j*, the data set *D_i_* was partitioned into a 90:10 ratio, and the larger set was used for training and the smaller set for development, and *D_j_* was the test set. In the case of *i* = *j*, the data set *D_i_* was divided into 3 parts in an 80:10:10 ratio, and the larger set was used for training and the 2 smaller sets for development and testing. Because the data set was small, and data bias was considerable, random oversampling was performed to ensure an equal proportion of the 4 labels.

#### Multi-subcorpus Incremental Learning

We predicted the labels of tweets *D_j_* mentioning drug query *K* = {*k_i_*} using the model *M_K_* built with tweets *D_K_* mentioning drug query *K*. *K* was the set of drug queries shown in the Methods section, containing 1 to 19 drugs, except drug query *j*. We divided the data set *D_K_* into 90:10 and used the larger set for training and 2 smaller sets for developing *D_K_* as the test set. We obtained the accuracy for the 20 drugs from this experiment and calculated the mean of the values. When adding the training data, we compared models trained using data chosen at random with models trained using data selected from those with similar structures. We defined *simX* as the result of a model trained with X drugs of similar structure and *rndX* as the result of a model trained with *X* drugs selected randomly.

### Drug Structure Similarity

To quantitatively calculate drug structure similarity, we used the Tanimoto similarity, which indicates the degree of similarity of chemical structures [36]. It was calculated by dividing the size of the product set of compound A and compound B fingerprints by the size of the sum set of compounds A and B. It is calculated as the percentage of bits in the substructure common to the 2 compounds.

To calculate the Tanimoto similarity, the chemical formula of each drug was converted into a simplified molecular input line entry system (SMILES) [37] to obtain the Morgan fingerprint vector. The radius size and the number of bits were set to 2 and 1024 bits, respectively.

### Ethical Considerations

This study did not require participants to be involved in any physical or mental intervention. As this research did not use personally identifiable information, it was exempt from institutional review board approval in accordance with the Ethical Guidelines for Medical and Health Research Involving Human Subjects stipulated by the Japanese national government.

## Results

### Single Subcorpus Transfer Learning

**Figure 2:**
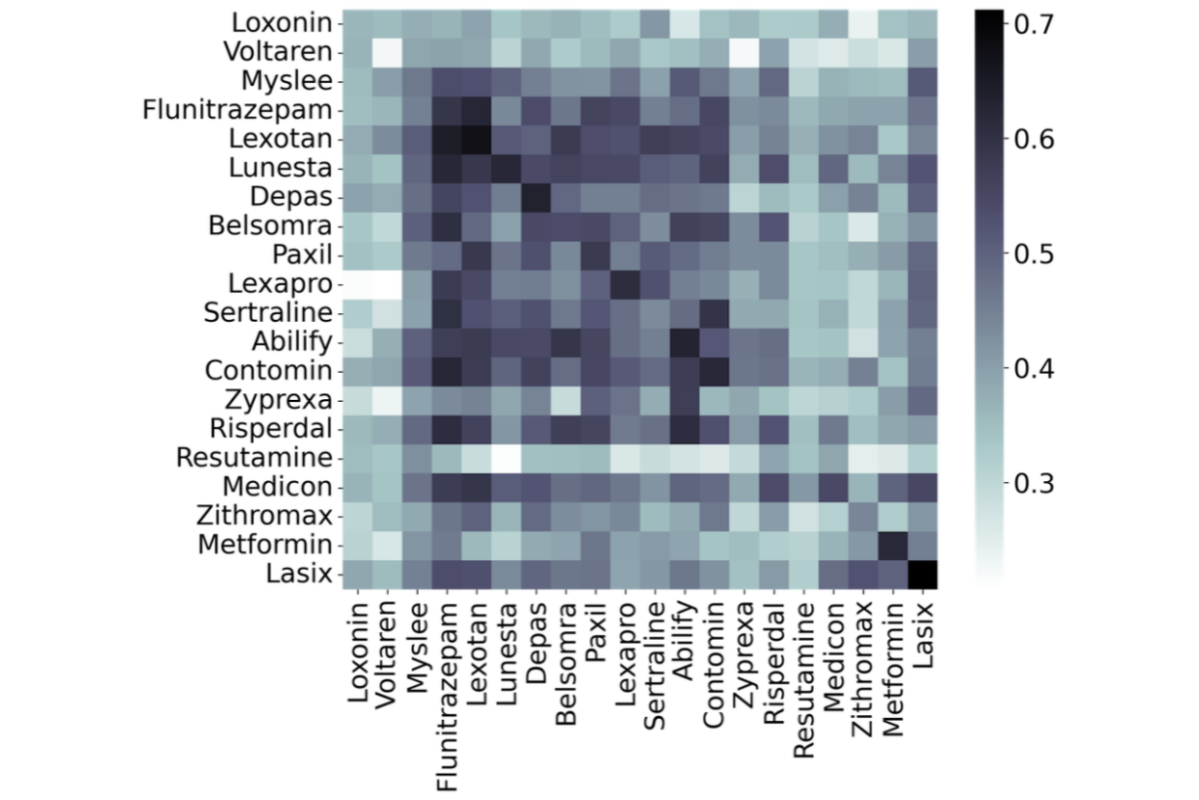
Value of F1 score for transfer learning.

The results of the validation using transfer learning are shown in Figure 2. The vertical and horizontal axes of the heatmap represent drug queries for the training and test data, respectively. The color intensity corresponds to the macro F1 values. The overall trend is that the values in the diagonal lines are the highest, indicating that learning using the corresponding query is the most efficient. However, *Myslee*, *Flunitrazepam*, *Lexotan*, *Depas*, *Belsomra*, *Paxil*, *Lexapro*, *Sertraline*, *Abilify*, *Contomin*, and *Risperdal* had darker areas that corresponded to the same drugs as well as the specific type of drugs. These drugs are classified into sleeping pills, anxiolytics, and antipsychotics. The darker colors of the areas suggest that tweets including these drug queries are available to each other for transfer learning, indicating a high possibility of transfer learning for drugs in similar categories.

**Figure 3:**
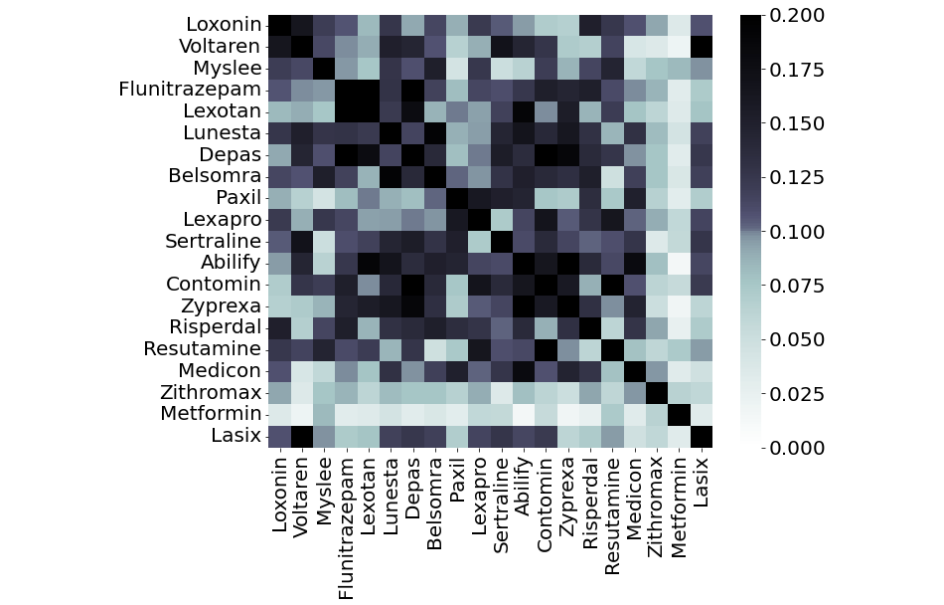
The Tanimoto similarity between each drug.

The Tanimoto similarity between the drugs is shown in Figure 3. This value is a numerical measure of the structural similarity of compounds, with a similarity of 1.0 for the same drug. Drugs used for similar purposes such as *Loxonin* and *Voltaren* are often structurally similar.

**Figure 4:**
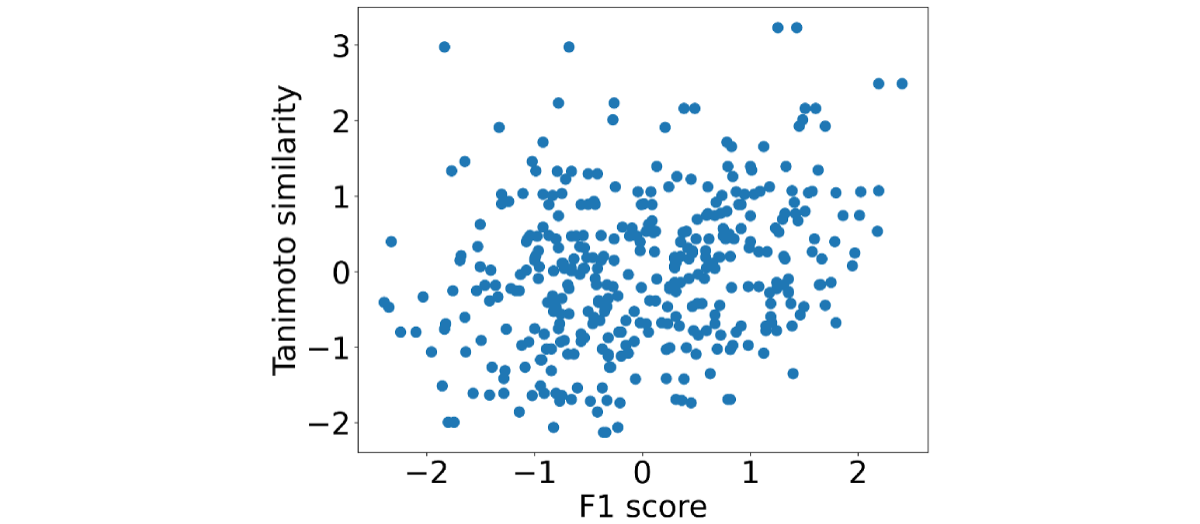
Relationship between the Tanimoto similarity and F1 value for each drug.

The relationship between the Tanimoto similarity and F1 values for each drug is shown in Figure 4. The vertical and horizontal axes were standardized with a mean of 0 and a variance of 1. The correlation between the Tanimoto similarity and the F1 value was 0.278 (P<.05). This result indicates that structural similarity is weakly correlated with the classification results.

### Multi-subcorpus Incremental Learning

The results of tweet classification by BERT and validation by transfer learning are presented in Table 1. The left panel of Table 1 (initial setting) shows the case where all data were used for training, whereas the right panel of Table 1 (transfer learning) shows the training results without data from the target drug query. *SimX* results from a model trained with X drugs of similar structures.

Table 1 shows that the *Rnd3* and *Sim3* results using 3 queries varied for each drug; however, *Sim3*, which was trained from drugs with high structural similarity, showed better overall values. Looking at each accuracy, the value of *Sim*3 for more drugs is 0.3 points higher than *Rnd3*, and the average value is higher. On the other hand, some values are higher for *Rnd1* than *Sim1*, even for randomly selected. This is due to the following factors. First, some drugs with different mechanisms have high structural similarity, such as *Voltaren* and *Lasix*, which have the highest structural similarity in this corpus. *Voltaren* is used as an antipyretic analgesic and *Lasix* is a prescription drug used as a diuretic. Thus, the textual properties are very different. The results of *Voltaren*, 0.618 for *Rnd1* and 0.454 for *Sim1*, show that the method of using a high-similarity drug for training does not work well. Second, even when drugs have other mechanisms and high structural similarity, selecting multiple drugs increases the likelihood that those with similar action mechanisms will be chosen. For example, drugs with high structural similarity to *Voltaren* include *Lasix*, *Sertraline*, and *Loxonin*. *Loxonin* is the same antipyretic analgesic, and adding *Loxonin* significantly improves the results (*Lasix*:0.454; *Lasix* + *Sertraline*:0.418; *Lasix* + *Sertraline* + *Loxonin*:0.634). Thus, selecting multiple drugs with high structural similarity implies that it is more likely that drugs with similar usage can be selected as training data rather than selecting a single drug.

**Figure 5:**
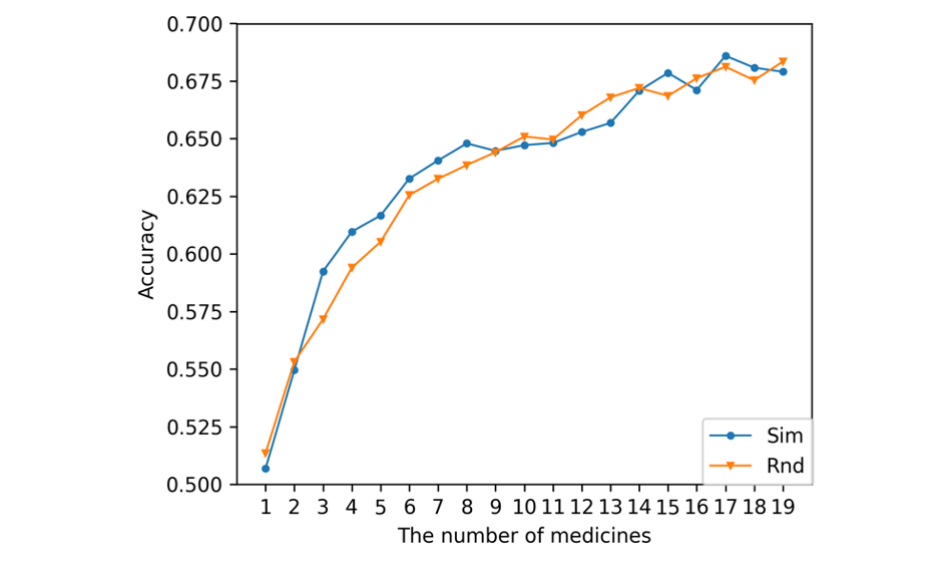
Comparison of the accuracy of the 2 models. *Sim* is a model transfer learned from a data set of drugs with close similarity; *Rnd* is a model transfer learned from a data set of randomly selected drugs.

Figure 5 shows a comparison of the accuracies of the 2 models. For *Sim*, the classification model using these similarities is trained by transfer with a data set created from drugs with close similarities. On the other hand, for *Rnd*, the model is trained by transferring a data set created from drugs selected at random. *Sim* showed better results than *Rnd* in the middle of the learning process; when approximately 10 drugs were added to the training data, there was no significant difference between the results learned randomly and the similarity.

**Figure 6:**
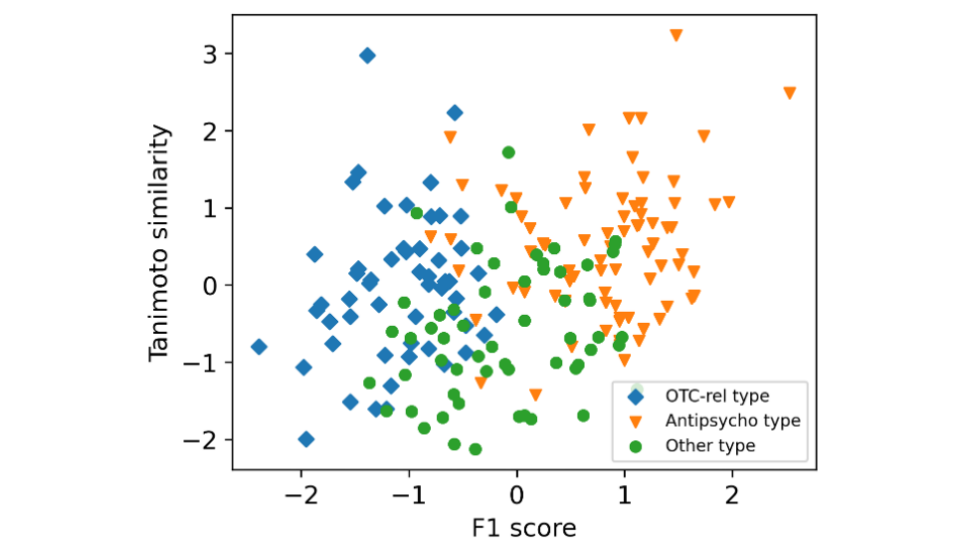
The Tanimoto similarity and F1 score pairs for each drug. *OTC-rel type* contains an over-the-counter (OTC) drug in one of the pairs; *antipsycho type* is a combination of antipsychotic medications such as sleeping pills, anxiolytics, and antischizophrenics; and *other type* is any other combination.).

Figure 6 shows the plot of each drug pair for each drug name. All plots are categorized into 3 major types: *OTC-rel type* contains an over-the-counter (OTC) drug in one of the pairs; *antipsycho type* is a combination of antipsychotic medications such as sleeping pills, anxiolytics, and antischizophrenics; and *other type* is any other combination.

**Table 1:** Comparison of initial setting and transfer learning.

	Initial setting, F1 score						Transfer learning, accuracy					
	NC-U/M^a^	NC-S^b^	G-u^c^	G-m^d^	Average	Rnd1		Sim1	Rnd3	Sim3	Rnd10	Sim10
Loxonin	41.7	0.0	76.1	45.3	65.6	64.8		63.6	60.6	*65.8* ^e^	63.3	64.9
Voltaren	50.0	33.3	77.3	65.7	71.0	*61.8*		45.4	48.8	*63.4*	66.0	66.1
Myslee	52.6	70.6	54.3	69.6	58.9	44.4		45.1	52.6	53.2	61.0	60.5
Flunitrazepam	44.4	96.0	69.2	84.5	73.3	53.5		*57.4*	65.3	66.5	69.0	68.8
Lexotan	66.7	100.0	77.7	60.6	74.1	42.5		*54.1*	61.9	61.3	68.2	69.1
Lunesta	53.3	75.0	67.4	58.5	63.1	54.4		52.8	58.6	57.8	66.2	68.2
Depas	64.0	93.3	75.2	71.7	74.4	45.8		*51.4*	50.0	*57.1*	59.9	59.4
Belsomra	54.5	0.0	73.4	74.6	70.5	*55.4*		51.2	61.1	*64.8*	64.1	66.3
Paxil	54.5	75.0	78.1	87.5	80.6	51.6		49.7	60.8	60.1	65.6	68.2
Lexapro	48.6	95.7	81.8	75.0	77.1	29.1		*58.8*	58.0	56.5	66.6	65.4
Sertraline	50.0	40.0	78.6	65.0	70.1	58.6		55.3	*64.6*	58.5	65.7	67.9
Abilify	46.7	100.0	78.3	64.9	71.3	56.9		*63.3*	62.5	*69.0*	74.7	72.3
Contomin	56.2	83.3	78.8	76.9	74.7	*51.0*		30.5	64.0	61.9	66.4	67.2
Zyprexa	16.7	0.0	67.3	71.4	63.0	53.9		*63.2*	53.7	*63.7*	66.7	67.3
Risperdal	48.0	80.0	60.0	71.0	63.0	56.2		57.5	62.0	59.3	70.4	69.6
Resutamine	72.2	0.0	65.1	75.5	71.1	*40.8*		33.5	36.2	*49.4*	51.7	51.6
Medicon	76.2	58.8	64.3	67.7	70.0	36.6		34.5	*49.6*	45.7	*52.0*	49.0
Zithromax	0.0	95.7	71.4	90.5	87.0	*62.2*		48.8	55.5	55.5	*75.7*	66.3
Metformin	47.1	88.2	66.7	93.7	87.3	60.0		71.4	69.2	*74.8*	74.4	78.8
Lasix	75.3	93.7	48.5	84.2	79.5	*47.7*		26.2	*48.4*	40.6	*54.3*	47.5
Average	59.7	86.4	73.1	76.6	72.3	51.4		50.7	57.1	*59.2*	64.7	65.1

^a^NC-u/m: noncompliant use or mention.

^b^NC-s: noncompliant sales.

^c^G-u: general use.

^d^G-m: general mention.

^e^Values individually at least 3 points higher than the corresponding value and averages at least 2 points higher than the corresponding value are in italics.

## Discussion

### Principal Results

This study observed that the learning efficiency in transfer learning is better for drugs with similar structures in a small corpus. Creating a large drug corpus is costly because it requires expertise and renewing the corpus because new drugs are often introduced. Therefore, the efficient usage of a small corpus is essential. A small drug corpus conveys information about the drugs themselves, such as their names and the structures of their active ingredients. Based on our findings, a drug-based metric, such as structural similarity, will contribute to model training, especially when resources such as corpora and budget are limited, such as in low-resource languages.

In Figure 6, *OTC-rel type* includes *Voltaren*, *Loxonin*, and *Restamine* in one of the pairs, and the F1 score tends to be lower overall. We hypothesize that the reason for the low F1 score is that pairs containing these drugs are less likely to have personal remote drug transactions classified as *NC-s*, and the tendency of their messages is different from that of prescribed drugs. In fact, OTC drug messages are more about individual transfers than remote drug transactions. Figure 6 shows the macro average of the F1 scores, but the scores of NC-s might lower the overall results. Additionally, the similarity tended to be relatively low. This is possibly because the analgesic drugs *Voltaren* and *Loxonin* and the antiallergic drug *Restamine* tend to have different structures than benzodiazepines and tricyclic antidepressants, which are the primary drugs selected in this study. Under the current experimental conditions, it is challenging to use transfer learning across prescription and OTC drugs.

*Antipsycho type* tends to have high F1 scores and similarity, possibly due to the similar textual properties and structures of antipsychotic drugs. The combination of these benzodiazepine sleep medications is the most common type of antipsychotic. Among antipsychotics, benzodiazepine sleep medications are most likely to be textually similar.

**Figure 7:**
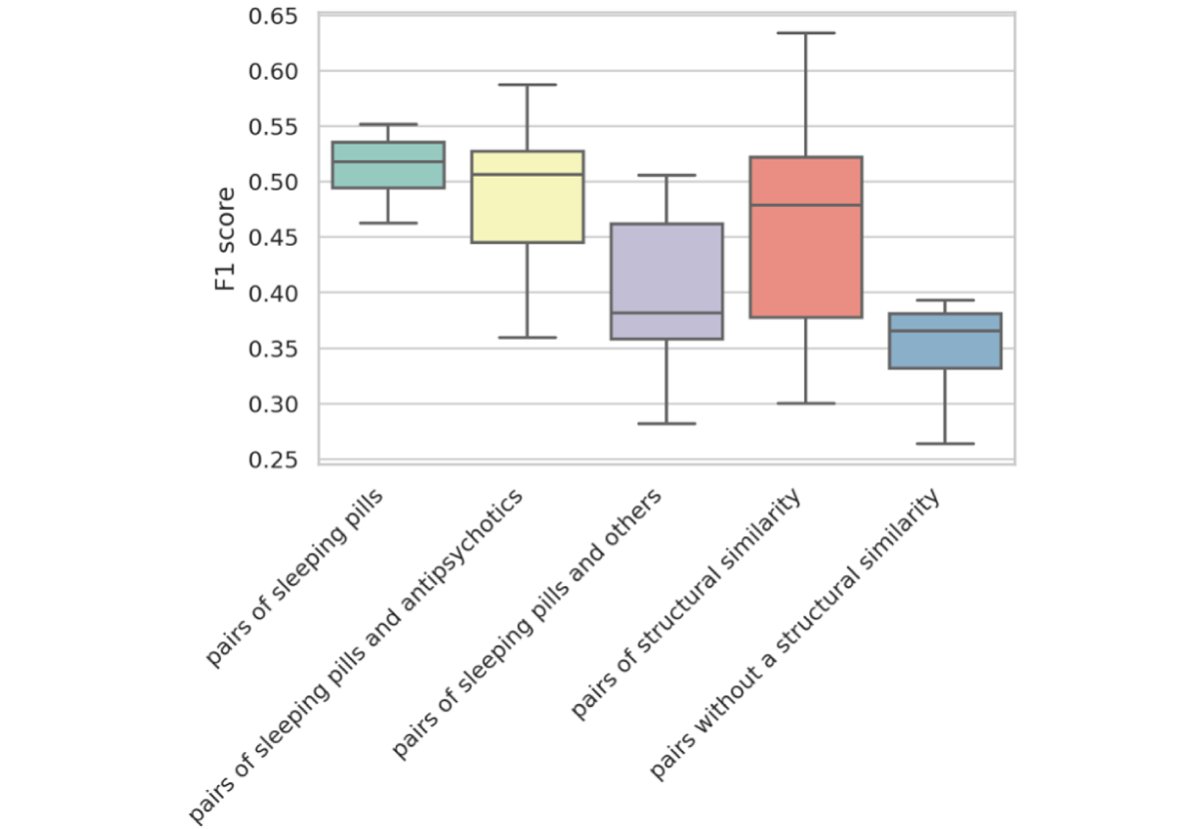
Comparison of the results of transfer learning for drugs with similar structure and drugs with similar indications.

Figure 7 compares the results of single-corpus transfer learning for drugs with similar structure and drugs with similar indications. In this figure, we visualize the results as pairs of sleeping pills as drugs with the same indication, pairs of sleeping pills and antipsychotics as drugs with similar indications, and pairs of sleeping pills and others as drugs with no similar indications. We also defined pairs of structural similarity as having a structural similarity greater than 0.15 and pairs without a structural similarity as having a structural similarity smaller than 0.03. As can be seen from this figure, the results of transfer learning are comparable for drug pairs with similar indications and drug pairs with high structural similarity. It also clearly shows the inefficiency of transfer learning for drugs with low structural similarity. These results indicate the usefulness of transfer learning by using structural similarity.

In our study, it is assumed that drugs with similar chemical structures can be used for similar purposes. This is based on the result demonstrated by Martin et al [30] that structurally similar drugs have similar mechanisms of action. The usage of drugs can also be considered similar. The similarity in usage means that the noncompliance of the drugs is similar and the texts are also similar. Through our study, we believe that we have shown that the structural similarity of drugs is useful for transfer learning of these textual classifications.

In addition, Jo et al [38] used deep learning to predict usage from SMILES transformed from chemical structures. Since most of the drugs selected in this study are antipsychotics classified as drugs for the nervous system, and they predicted several uses of drugs, including the nervous system, with about 90% accuracy, better results could be obtained by using models that can handle structural information in more detail, such as deep learning models, rather than just simple similarity.

**Figure 8:**
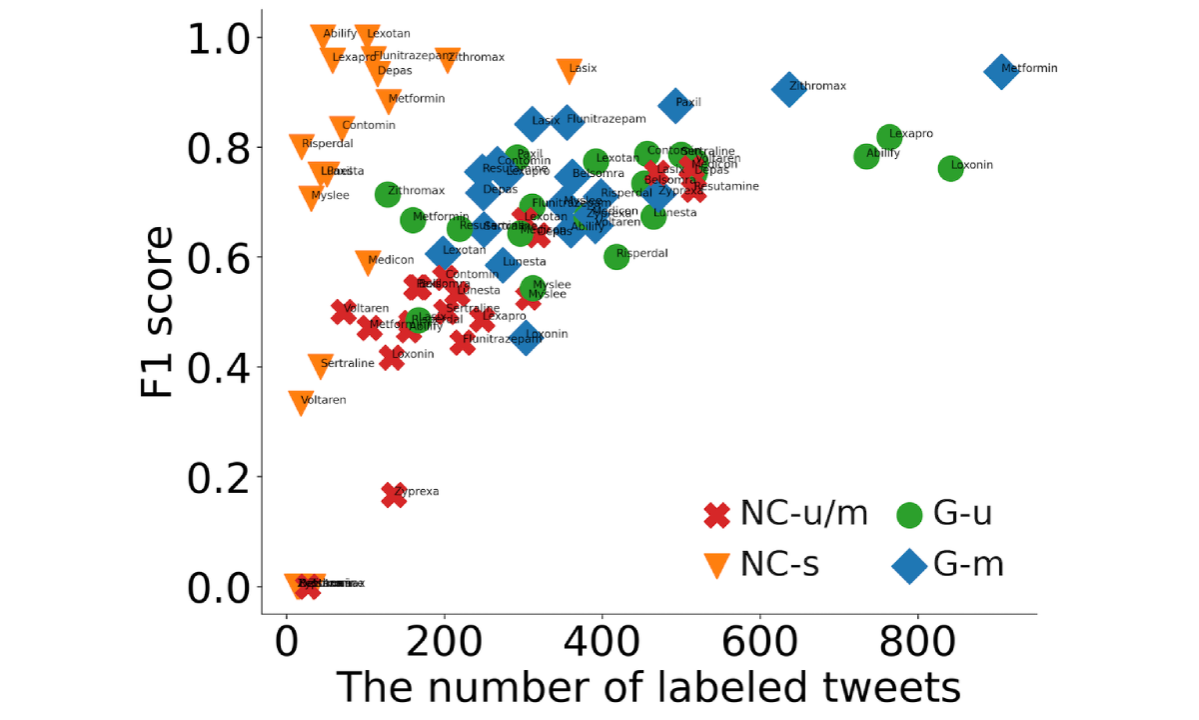
Scatterplot showing the relationship between the number of tweets and F1 value for each drug. G-m: general mention; G-u: general use; NC-s: noncompliant sales; NC-u/m: noncompliant use or mention.

Figure 8 plots the relationship between the number of labeled tweets and the F1 value for each drug in the corpus, indicating that the F1 value increases with the number of tweets. On the other hand, the F1 scores of *NC-s*, unlike the different categories, do not depend significantly on the number of tweets, and tweets classified as *NC-s* are similar in content, even if the type of drug mentioned differs. The F1 score for categorizing a tweet as abuse was 0.53 [29], which is considered adequate. The overall F1 score was 0.723, which is also a favorable result compared to those in previous studies [29]. Since the F1 score reached its peak when the number of tweets with the corresponding label reached approximately 500, this inferred that 500 tweets are one of the guidelines when preparing training data for each query.

### Limitations

In this study, the experiments were conducted using only 20 different types of drugs. The categories of drugs included analgesics, sleeping pills and anxiolytics, antipsychotics and antidepressants, antiallergics, antitussives, antibiotics, antidiabetics, and diuretics. Not all types were covered; expansion of the drug category is a significant issue for the future. Additionally, most drugs were categorized as antipsychotics. This bias may have affected the study results.

The relatively low interannotator agreement limited the performance of the models. Annotation schemes could be improved to obtain better metrics. Furthermore, the correlations did not necessarily indicate any higher-level associations between structural similarity and metrics from the results.

We only used the Tanimoto similarity as the structural similarity without considering the 3D structure. Considering that the action of the mechanism was based on the 3D structure, calculating the similarity with the 3D structure can be improved. A detailed investigation of this learning method is required.

### Conclusions

In this study, we assessed the usefulness of the structural similarity of drugs by using a corpus annotated with medication noncompliance. It was found that structural similarity can be used for more efficient learning of training data with a limited number of drugs. On the other hand, using a corpus in the case of a new drug introduction or learning in a low-resource language with a small corpus, it is possible to provide a guideline for using training data from drugs with a similar structure. We believe that this can provide a procedure for training data for learning in low-resource languages where the differences are slight, and the corpus is limited.

## Appendix

Multimedia Appendix 1 Annotation guideline of MediA corpus.

## Abbreviations

ATC: Anatomical Therapeutic Chemical
BERT: bidirectional encoder representations from transformers
G-m: general mention
G-u: general use
NC-s: noncompliant sales
NC-u/m: noncompliant use or mention
OTC: over-the-counter
SMILES: simplified molecular input line entry system

## Data Availability

The data sets generated or analyzed during this study are available [39].
