# Supplementary material for: Transferability Based on Drug Structure Similarity in the Automatic Classification of Noncompliant Drug Use on Social Media: Natural Language Processing Approach
Source: J Med Internet Res. 2023 May 3;25:e44870. doi: 10.2196/44870 (PMC10193216; doi:10.2196/44870)
Supplement: Multimedia Appendix 1 [file jmir_v25i1e44870_app1.pdf]

Corpus Guidelines

1. Annotation Categories

- NONCOMPLIANT USE/MENTION (NC-U/M): Noncompliance falls into this category. This occurs when the speaker has an inappropriate understanding of the drug. Inappropriate understanding refers to the misunderstanding about the medication or its dosage, such as not following the dosage specified on the package insert, not following the doctor's instructions, and receiving more medication for treatment than originally required. Even without these behaviors, if the originator of the tweet is considered to have an inappropriate understanding, the tweet is identified as "Noncompliant use/mention (NC-u/m)."
- NONCOMPLIANT SALES (NC-S): This category specifies noncomplaint statements about the sale and purchase of medicines.
- GENERAL USE (G-U): People who take any medication not included in the "NC" categories are classified in this category.
- GENERAL MENTION (G-M): If the statement is not included in any of the other categories, it is categorized into General mention (G-m).

2. Basic Criteria

To determine whether a person has an inappropriate "Understanding" of a drug, we focus on the understanding that can be inferred from the speaker's own statements. If the statement suggests inappropriate understanding, the tweet is classified as "NC." The Tweets categorized as "NC," they are categorized as either "NC-s" or "NC-u/m" according to the contents described in the corpus classification. Tweets that are not categorized as "NC" are categorized as "G-u" or "General mention (G-m)" according to whether medication is being consumed.

3. Categorization Guide

To make the categorization clearer, we created detailed categorization examples (subcategories). Tables 1, 2, 3 and 4 show that each example is categorized so that it can be used as a basis for determining classification. All examples are originally written in Japanese.

- Noncompliant use/mention (NC-u/m)
  - Inappropriate dosage or frequency: This is when the dosage is determined to be higher or lower than the prescribed dosage or frequency. This includes cases where the patient stops taking the medication at their own discretion.
  - Expressions implying abuse: A case in which expressions implying abuse are included. In Japanese, "nomu (drink)" and "hukuyakusuru (take medication)" are commonly used verbs for taking medication, but other verbs such as "ireru," "kimeru," "poriporisuru," and "shitai" imply abuse.
  - Mistake in taking medication: A case in which an expression indicating a mistake in consuming medication is included.
  - Excessive possession: A case of prescribing evidently more than required.
  - Dosing with other than water or tea: Anything apart from water or tea is considered inappropriate use because it increases the possibility of drug interactions.
  - Expressions of tolerance: The expression "developed tolerance" may have been used inappropriately for a long time.
  - Mental status: Expressions of dependence on medication may be owing to inappropriate or inappropriate use of the medication.
  - Misinterpretation of medicine action: If a person misinterprets the action, he or she is considered to have an inappropriate perception.
  - False intent: This is a case in which the drug is consumed with the intention of having an effect that is not the intended effect. The intended action should be in accordance with the package insert.
  - Wrong storage conditions: Poor storage conditions also fall under the category of inappropriate use, as it is considered that the medicine that should be consumed is not being consumed.
- Noncompliant sales(NC-s)
  - Online sales: This is the case in which drugs are sold online.
  - Online purchase: This is the case of purchasing medicines. It is also included in the case of intent.
  - Transfer: This is the case in which the drug is transferred.
- General use(G-u
  - Use by the individual: This is when the individual is using the drug. Even if the tweet lacks the expression of consuming the drug, the tweet is categorized as use when it implied that the person is taking the drug such as they obtains prescribed medicine in a hos-

pital visit.

- General mention (G-m)
  - Correct understanding of efficacy: Whether the tweet has no problem in understanding of efficacy, it is basically judged by the efficacy by comparing it with the action section of the package insert.
  - The person's past dose: If it can be inferred that the person used to consume medicines in the past but not now, the tweet is General mention (G-m).
  - General information: Other information about common medicines such as news.
  - No meaning: A case in which the available information makes no sense.

Table 1: Subcategories and examples of NC-u/m

Subcategories	Examples
Inappropriate dosage or frequency	1シートのもう<br>I will take 1 sheet<br>*デパス* 多めに飲んだ<br>I took more *Depas*
Expressions implying abuse	私も *メジコン* いれたんだけど<br>I “ireru” *Medicon*, too<br>*ロキソニン* ぽりぽりしたい<br>I want to “poriporisuru” *Loxonin*
Mistake in taking medication	*ロキソニン* 飲むつもりだったのに、*ボルタレン* 飲んじゃった<br>I was going to take *Loxonin*, but I accidentally took *Voltaren*<br>さっき間違えて *ルネスタ* 2 個飲んじゃったんだけど<br>I took two *Lunesta* by mistake earlier
Excessive possession	余りまくた。 *デパス* ！ *レキソタン* ！ *マイスリー* ！<br>I’ve got a surplus. *Depas*! *Lexotan*! *Myslee*!<br>*レクサプロ* しか飲んでないふりしてるけど沢山余ってる<br>I pretend I’m only taking *Lexapro*, but I have a lot left over
Contraindications and precautions for co-administration	お酒飲んでも *デパス* 飲んでも全然眠れない<br>I can’t sleep at all, even after drinking alcohol and taking *Depas*.<br>薬 ストラテラ40mg、 *レクサプロ* 10mg、 *エビリファイ* 3mg<br>Medications: Strattera 40mg, *Lexapro* 10 mg, *Abilify* 3 mg
Dosing with other than water or tea	*ロキソニン* を胃に悪いコーヒーで飲む<br>Take *Loxonin* with coffee, which is bad for your stomach<br>今粉の *エビリファイ* をコーラで飲もうとしたら<br>I just tried to drink powdered *Abilify* with Coke
Expressions of tolerance	ダメだ *デパス* 完全に効かなくなった<br>No, *Depas*, it’s completely stopped working<br>*フルニトラゼパム* 2㎎効かなくなってるぅぅ<br>2 mg of *Flunitrazepam* has stopped working.
Mental status	眠剤と *デパス* に依存症になって<br>I became addicted to sleeping pills and depressants<br>*デパス* の処方やめるって言われたら生きていかれないと思う<br>I don’t think I could live with myself if they said they were going to stop prescribing *Depas*
Misinterpretation of action	*ロキソニン* は胃に優しいので<br>*Loxonin* is gentle on the stomach.<br>口内炎痛すぎて *ロキソニン* 飲んだら 胃が荒れ模様〜〜??<br>I took *Loxonin* because of the painful mouth ulcers and it made my stomach upset...??
False intent	カー！！ *デパス* 効いてきてふわふわ気持ちいい<br>I’m feeling fluffy and fluffy now that my *Depas* is working<br>*ラシックス* のんで47.6〜あーやっとでこの体重かよ<br>I’ve been taking *Lasix*, and I’m at 47.6, so I’m barely at this weight
Wrong storage conditions	明日病院だが、なぜか *レクサプロ* だけ今日の分無いんだよなー<br>I’m going to the hospital tomorrow, but I don’t know why I don’t have *Lexapro* for today<br>モーラスは見つかるのに、*ロキソニン* は見つからない<br>I can find Morus, but I can’t find *Loxonin*

Table 2: Subcategoris and examples of NC-s

Subcategories	Examples
Online sales	*レクサプロ* ・ジェネリック抗うつ剤の *レクサプロ* ジェネリック医療品でうつ病や、パニック障害、対人恐怖症、不安障害に有効的です 20mg×200錠 ¥14,000⇒¥11,950 15%割引... *Lexapro* generic antidepressant *Lexapro* generic medical product is effective for depression, panic disorder, anthropophobia, and anxiety disorders 20 mg x 200 tablets ¥14,000⇒ ¥11,950 15% discount... 初めまして、 *デパス* 1mg可能です。 DMください Nice to meet you. 1 mg of *Depas* is available. Please DM me
Online purchase	#お薬もぐもぐ # *マイスリー* #ハルシオン # *デパス* 上記二つ優先で探してます。 安価でアプリ経由で譲ってくれる方いらっしゃいましたら反応御願いします。 I'm looking for the above two priorities: #drug mugging #*Myslee* #*Halcion* #*Depas* Please respond if you are willing to sell them via the app at a low price. 利尿剤の *ラシックス* 買ってみよっかなー I think I'll buy some *Lasix*, a diuretic.
Transfer	*レクサプロ* 送ろうか？ Do you want me to send *Lexapro*?

Table 3: Subcategories and examples of Genaral use(G-u)

Subcategories	Examples
Use by the individual	*リスパダール* 飲んだ〜ぞ。明日絶対起きる I took *Risperdal*. I'm definitely going to wake up tomorrow 私は *コントミン* がいまいち効かない I can't seem to get *Contomin* to work for me 呼吸は *レキソタン* や *デパス* があるんですが、、 安静にしてようと思います I have *Lexotan* and *Depas* for breathing, but I think I'll just rest

Table 4: Subcategories and examples of General mention (G-m)

Subcategories	Examples
Correct understanding of efficacy	*ラシックス* は心臓病とか胸水腹水とか"病気"の時に飲むものだし健康な時に飲むものじゃないよ...。 *Lasix* is for people with heart disease, pleural effusion, ascites, and other "illnesses", not for people who are healthy....
The person's past dose	私は *エビリファイ* の方が寝たきりでした。外出も辛かったです。 I was more bedridden on *Abilify*. It used to be hard for me to go out 私は以前飲んでたのは *ロキソニン* ガスロン、メチコバールだった！ I used to take *Loxonin*, Gaslone, Methycobal
News	*メトホルミン* 含有製剤のNDMA分析を指示 #日経メディカル NDMA analysis of *Metformin*-containing drugs ordered Nikkei Medical
No meaning	どうせなら *デパス* ではい青くしたい所やった I would've taken *Depas* to turn "hai" blue
